# Influence Mechanism of Dynamic Evolution of Chinese Entrepreneurs’ Entrepreneurial Motivation on Performance—The Role of Turning Points and Empathy

**DOI:** 10.3389/fpsyg.2020.474044

**Published:** 2020-12-29

**Authors:** Yiqi Zhao, Xianfeng Zhao, Yuanjian Qin

**Affiliations:** ^1^School of Economics and Management, Shijiazhuang Tiedao University, Shijiazhuang, China; ^2^School of Management, Hebei GEO University, Shijiazhuang, China; ^3^School of Management, Wuhan University of Technology, Wuhan, China

**Keywords:** entrepreneurial motivation, turning points, Empathy, egoist motivation, altruistic motivation

## Abstract

Using the Grounded theory, we took 15 Chinese entrepreneurs as the research objects and constructed the entrepreneurial process model of dynamic evolution of entrepreneurial motivation. The model includes seven themes, such as egoist motivation, bottleneck, altruistic motivation, TP/MTP, empathy, responsible leadership, CSR implementation and entrepreneurial performance. Through the analysis of the internal relations between these elements, we abstracts the law of the dynamic evolution process of entrepreneurial motivation of Chinese entrepreneurs, and reveals the mechanism of the dynamic evolution process of entrepreneurial motivation. The theoretical contribution of this paper is mainly reflected in the following two aspects: (1) it enriches and expands the research results in the field of entrepreneurship motivation of Chinese entrepreneurs. From the perspective of entrepreneurial motivation, this study analyzes the dynamic evolution process of Internet entrepreneurs’ entrepreneurial motivation, extracts the rules of entrepreneurial process of dynamic evolution of Internet entrepreneurs, and provides a new path for enriching and expanding the research on Internet entrepreneurs’ entrepreneurial motivation. (2) The study of turning point (TP) that enriches and complements the dynamic evolution of entrepreneurs’ entrepreneurial motivation. Using grounded theory, this paper deeply analyzes the reasons for the dynamic evolution of entrepreneurial motivation, and provides empirical evidence for the research on the evolution of localized entrepreneurial motivation in China.

## Introduction

In recent years, the public entrepreneurship, innovation is booming, has given rise to numerous market new forces (“The state council on strengthening peoples innovation-driven development strategy to further advance the public undertaking development innovation opinion,” 2017), the results of the venture entrepreneurs tend to have three, direct entrepreneurial success, through one or more failures startups succeed, finally a is still in the start-up failure (Zhao et al., 2014). In the context of “Internet+,” China supports “Internet+” entrepreneurship and innovation, giving full play to the supporting role of the Internet in entrepreneurship and innovation(The state council on promoting action guidance for “Internet+,” 2015). Compared with traditional enterprises, Internet + has endowed the industry with more possibilities, accelerated the ability of enterprises to connect users and serve users, and accelerated the speed of change of enterprises (Chen, 2017). Compared with traditional enterprises, successful start-up of Chinese enterprises requires entrepreneurs to have stronger ability to deal with environmental uncertainty and accelerate technology iteration (Chen, 2017).

Scholars have classified entrepreneurial motivation from different perspectives, and most studies agree that economic benefits and self-interest are the most prominent motivations for for-profit enterprises (Schumpeter, 1934; Carsrud and BraNnback, 2011), and the motivation to benefit the society usually only promotes the establishment of non-profit enterprises (Nga and Shamuganathan, 2010), but many for-profit organizations also create a lot of social value (Tan et al., 2005; Phills et al., 2008; Acs et al., 2013). Existing studies on the entrepreneurial motivation of commercial entrepreneurs are divided. Research found that in some cases major events restructure a person’s values and even sublimate values (Hemingway and Starkey, 2018). In the life course of entrepreneurs, is there an event that changes the motivation of entrepreneurship? Although TP has been studied in developmental psychology (for example, Gotlib and Wheaton, 1997; McAdams et al., 2001; Pillemer, 2001) and has conducted limited research in the leadership literature (for example, Bennis and Thomas, 2002; Shamir and Eilam, 2005; Janson, 2008; Ligon et al., 2008; Albert and Vadla, 2009). Based on this study, we intend to classify and summarize TP from the perspective of dynamic evolution of entrepreneurial motivation. Empathy refers to the emotional “tendency to. Feeling warmth, compassion, and caring about negative experiences that others have had”. Major events trigger entrepreneurs to empathize with specific groups of people and may turn into pro-social intentions (Johanna and Jeffrey, 2006), which in turn leads to the evolution of entrepreneurial motivation. Research on how the evolution of existing entrepreneurial motivation affects the entrepreneurial process is rare.

This study divides entrepreneurial motivation into self-interest motivation and other-orientation motivation from a cognitive perspective, and explores the connotation, dimensions and levels of entrepreneurial motives of Chinese entrepreneurs. The motivation for self-interest is divided into material and achievement motivation. Other-orientation motivation is divided into social responsibility and social mission. Taking 15 successful Chinese companies as examples to conduct multi-case research, summarizing the triggering events (TP) of entrepreneurs’ motivation dynamics, and extracting the reasons and the entrepreneurial process rules of dynamic evolution of Chinese entrepreneurs’ entrepreneurial motivation. The theoretical contributions of this paper are mainly reflected in the following two aspects:(1) enrich and expand the research results in the field of entrepreneurial motivation of Chinese entrepreneurs. (2) it enriches and complements the study on the triggering events (TP) of the dynamic evolution of entrepreneurial motivation.

## Literature Review

### Entrepreneurial Motivation

In the existing research, the research on entrepreneurial motivation of commercial entrepreneurs mainly focuses on internal motivation and extrinsic motivation (Deci and Ryan, 1985; Amabile, 1996). The intrinsic motivation shows the individual’s deep interest in tasks or work enjoyment and passion, entrepreneurs driven by intrinsic motivation want to get, that is, rewards, goals, reputation and recognition (Ryan and Deci, 2000). Extrinsic motivation refers to desires derived from external stimuli, such as wealth (Birley and Westhead, 1994), financial success or good job (Ryan and Deci, 2000; Carter et al., 2003). It can be seen that existing research has identified motivations for achievement, independence, wealth and status, which are the motivation for entrepreneurship (e.g., McClelland, 1961; Jenssen and Kolvereid, 1992; Collins et al., 2004; Benzing et al., 2009). These are all categories of self-interest motivation. Self-interest motivation is a state of motivation whose ultimate goal is to increase one’s own welfare (Batson, 2008). Most studies agree that economic gains and self-interest are the most prominent motives for for-profit companies (Schumpeter, 1934; Carsrud and BraNnback, 2011), and the motivation to benefit society usually only drives the establishment of non-profit companies (Nga and Shamuganathan, 2010). However, some for-profit organizations create economic and social value (Tan et al., 2005; Phills et al., 2008; Acs et al., 2013). Alcantara and Kshetri (2013) studied the impact of other-orientation motivation on performance for Japanese entrepreneurs. But like all single-country studies, results based on Japanese entrepreneurs may not be extended to other countries. Is creating social value (altruistic motivation) an entrepreneurial motive for Chinese entrepreneurs?

### Evolutionary Reasons of Entrepreneurial Motivation

Research has found that in some cases, major events can reconstruct one’s values, and even sublimate them (Hemingway and Starkey, 2018). Once the moral characteristics are strongly triggered, they will become the stable character characteristics of the triggered person (Hemingway and Starkey, 2018). It is hoped that management education will gradually start to make an impact in this field, by empowering individuals to rely on their own reasonable judgment and good intuition and emotion to promote questions about how to develop character and ideas to pursue moral life (Akrovou and Sison, 2016). Hemingway and Starkey (2018) found in their research that leaders will change their values due to some major trigger event—Turning Point, from self-promotion values to self-transcendence personal values and become responsible leaders. Self-transcending personal values focus on the welfare and interests of others (benevolent and universalist values), while self-improving personal values focus on self-interest and dominance over others (Schwartz, 2010). Hemingway and Starkey (2018) analyzed the turning point (TP) in the role of responsible leadership development, and the TP as corporate social responsibility (CSR) a mechanism for the development of leadership, and through the exploratory ethnography method the TP to be classed as experience in the workplace, inspirational education experience, religious Epiphany and serious family disease/bereavement. Then, will the entrepreneurial motivation of entrepreneurs change due to some major events? In the context of China, will it add new dimensions to the research of TP?

### Empathy

Empathy refers to the cognitive process in which individuals adopt the opinions of others in an attempt to understand the preferences, values, and needs of others (Parker and Axtell, 2001). When entrepreneurs have empathy, their desire to benefit others causes them to pay high attention to others’ situation and ideas (Galinsky et al., 2006). According to relevant studies on motivational information processing theory, when entrepreneurs think from the perspective of others, they tend to consolidate and adjust all viewpoints in an integrated way of thinking (De Dreu et al., 2000). And when entrepreneurs consider more different perspectives, they will have a deeper understanding of the perspectives from different groups (such as various stakeholders) and ultimately choose ideas that are beneficial to these groups (Amabile, 1996). Numerous studies in psychology and management have shown that individuals with prosocial motivation are more likely to adopt the views of others (such as colleagues, superiors, suppliers, clients) (Batson et al., 1997; De Dreu et al., 2000; Parker and Axtell, 2001; Axtell et al., 2007).

## Research Design

### Methods

Due to the complexity and dynamic nature of entrepreneurial process, qualitative research method is suitable, and we choose grounded theory. This method is a qualitative research method based on empirical data and top-down construction theory proposed by Strauss and Corbin (1997). The basic analysis steps are: data collection, Open coding, Axial Coding and Selective Coding. Atlas.ti 8.0 software was used for open coding. This paper adopts the method of multi-case longitudinal research. Although the process is complicated and difficult, it can follow the logic of “differential replication” to better expand and repeatedly verify the research conclusions (Brown and Eisenhardt, 1997). The main reasons are: (1) The current research on entrepreneurial motivation and life history is based on entrepreneurial experience in Western countries (Jayawarna et al., 2013), it is necessary to study entrepreneurial motivation and life course in Chinese context; (2) The dynamic evolution of entrepreneurial motivation is a complex and multi-dimensional situation. The recovery process is not the same. The summary of the single case process can not reflect the common characteristics. It is necessary to summarize the experience of different entrepreneurs to start a business, and it is possible to draw general rules from it. Practice activities play a guiding role; (3) The time horizon of the dynamic evolution process of entrepreneurial motivation is uncertain, statistical data is difficult to “just happen,” and long-term tracking case studies are more feasible (Chen et al., 2019).

### Case Selection

Berg and Lune (2017) proposed 3–7 best case studies for multiple case studies. According to the principle of theoretical sampling (Claser and Strauss, 1967) 17, select Chinese entrepreneurs to conduct research, the specific criteria are as follows. The sample screening criteria are: (1) The company founded is an Chinese company; (2) entrepreneurship time is more than 3 years; (3) According to the growth cycle of the company, choose enterprises that are in the growth stage and are in the leading position in the industry. According to the above selection principle, this study selected 15 entrepreneurial companies as research samples. In order to protect the privacy of entrepreneurs and information on entrepreneurial enterprises, entrepreneurs are replaced by English letters in the text. The basic situation is shown in Table 1.

**TABLE 1:** Case description.

Entrepreneur	Description
1	Fitness app with social attributes
2	Online English teaching for children
3	Short video app
4	Network Knowledge Quiz APP
5	Internet hospital platform of online diagnosis and treatment
6	Smart electric car
7	Online management and e-business services
8	High-end two-wheeled electric vehicle manufacturing enterprises
9	Pharmaceutical retail chain
10	Wedding photography
11	Direct beauty chain
12	Medical and health food production enterprises
13	Property service enterprises
14	Comprehensive operators of aged care services
15	Manufacturers of leather shoes and leather products

### Data Collection

In order to make the conclusions of the research accurately reflect the facts, this paper collects second-hand data through various methods. The second-hand data mainly includes: (1) Check the entrepreneur’s resume through the network channels such as Tianyancha, corporate official website and Baidu; (2) Tencent University’s “CEO is coming” program interviews video interviews, each of the entrepreneurs in the case has an hour of interviews, interviews involving entrepreneurs’ personal growth experience, entrepreneurial initials, entrepreneurial process, entrepreneurial bottlenecks, Entrepreneurial experience, etc. (3) Interview press releases related to the founder, and articles related to the founder published by the enterprise public account; (4) Corporate social responsibility report, network information, industry information, news reports, professional database documents, public company documents, etc.

### Data Analysis Strategy

In order to ensure the accuracy and variability of the research conclusion, this study followed the data analysis procedure suggested by Strauss and Corbin (1997) to carry out the category induction and model construction of the entrepreneurial behavior process of returnees:

(1) Establish a coding team. In order to avoid the impact of the researcher’s subjective bias on the coding results and reduce the error of the research results, the first author of this paper and two other graduate students in the direction of entrepreneurship and management formed a coding team. The first author conducted theoretical and technical training for the other two members of the group, and then each of them was responsible for conducting trial coding on a case subject, and discussed the coding results to find problems in the coding process and propose solutions. After that, the team members coded the six case subjects separately, and when they had different opinions, they discussed together until the coding results were consistent.
(2) Build the coding database. Use Excel to create a form for each case object to record the whole detailed coding process, and the modification in the coding process is also faithfully recorded in the Excel sheet.
(3) Continuous comparative analysis of object coding process of different cases. For comparative analysis is one of the core idea of grounded theory, it requires the simultaneous data collection and data analysis, the researchers once found new problems when collecting information from other sources to find new information to verify, constantly formed by using the new information and according to the existing data category comparison, through the theory of “the collection of data—formation—to collect data—fixed perfect theory” the cycle of process to construct the theoretical model.

## Data Analysis

### Open Coding

Open coding is the purpose in the process of preliminary analysis of the data to identify phenomena, defining the concept, and then found that category, which requires researchers remain completely open, the data coding, layered conceptual and abstract line by line, by constantly breaking up the concept of the data and abstracting, ultimately determine the concept of category. Following the open coding process of “defining phenomenon—developing concept—exploring category,” the open coding of data in this paper is shown in Table 2. (1) Labeling, that is, some of the database may be related to the entrepreneurial motivation, entrepreneurial process statements; (2) define the phenomenon, that is, the label for simplification and preliminary refinement; (3) conceptualization, that is to reclassify the phenomenon, the concept of classification; (4) category, that is to the relevant concept of abstraction, classification. After open decoding with ATLAS.ti 8.0, 21 subtheme and 5 themes describing entrepreneurial motivation and entrepreneurial process were finally obtained.

**TABLE 2:** Themes and subthemes.

Themes	Subthemes
1. Egoistic motivation	1.1 Material motivation
	1.2 Personal values and inner satisfaction
	1.3 Risk-taking propensity
	1.4 Grow orientation
2. Bottleneck	2.1 Pain point
	2.2 Business losses
	2.3 Management problems
3. TP/MTP	3.1 Life events in the present
	3.2 Life events in the past
	3.3 Awareness of unmet social needs
	3.4 Ideology
4. CSR implementation	4.1 CSR implementation to employee
	4.2 CSR implementation to society
	4.3 CSR implementation to customer
	4.4 CSR implementation to supplier
5. Empathy	5.1 Empathy to employee
	5.2 Empathy to customer
	5.3 Empathy to society
	5.4 Empathy to supplier
6. Altruistic motivation	
7. Performance	

### Axial Coding

**FIGURE 1:**
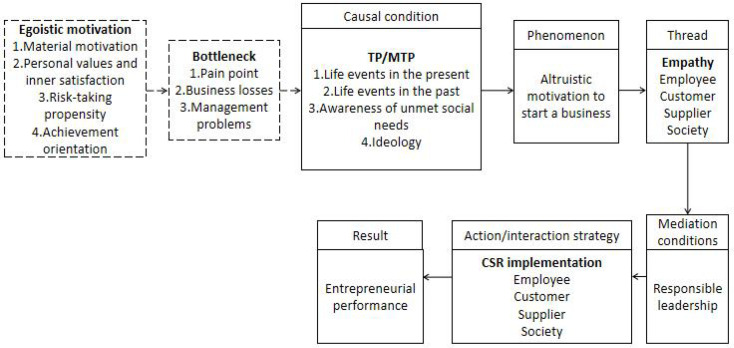
Entrepreneurial motivation evolution canonical model of Chinese entrepreneurs.

According to the categorical variables obtained by open coding, using “causal condition—phenomenon—context—intermediary condition—action/interaction strategy—result,” this paper takes entrepreneur 1 as an example to get the logical relationship of its entrepreneurs’ entrepreneurial process, and on the basis of getting the basic process, continues to compare and analyze the follow-up cases, so as to ensure that the theory reaches saturation. In this study, by the 11th case, the theoretical saturation has basically been reached, and no new dimension has appeared. The last four cases are used to verify the theoretical saturation, which finally proves that the model has good saturation. The logical relationship diagram of the entrepreneurial process of entrepreneurs in 15 cases is shown in Figure 1 below.

### Selective Coding

**FIGURE 2:**
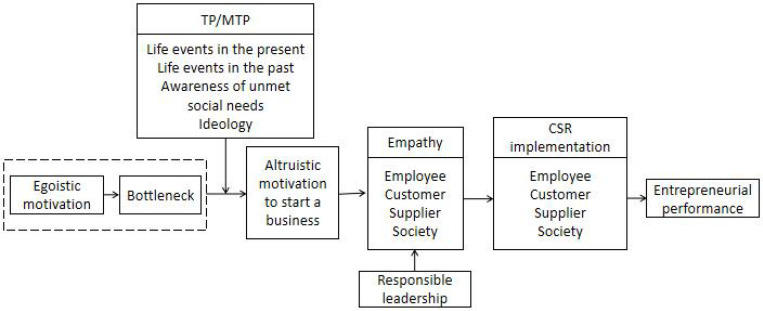
Entrepreneurial motivation evolution process model of Chinese entrepreneurs.

The above open coding and axial coding show the basic framework of entrepreneur A’ entrepreneurial motivation and entrepreneurial process. In order to further understand the mechanism of Chinese entrepreneur’ entrepreneurial process, selective coding is also needed to carry out in-depth analysis and discussion. Selective coding is the process of relating certain core categories to other categories, verifying their relationships, and supplementing the conceptualization of yet-to-be formed categories. Through selective coding, a relatively clear story line can be obtained: (1) Some entrepreneurs’ initial entrepreneurial motivation is egoistic motivation, including material motivation, personal values and inner satisfaction, risk-taking propensity or achievement orientation. (2) These entrepreneurs may encounter business losses, management problems, industry pain points and other bottlenecks in the process of starting a business. (3) As a causal condition, entrepreneurs change their moral values because of life events in the present, life events in the past, awareness of unmet social needs, or ideology. (4) Establishing or operating an enterprise with altruistic motivation is the phenomenon caused by TP/MTP. (5) Empathy for customers, employees, suppliers and society occurs during the process of transforming entrepreneurial motivation into altruistic motivation. Empathy is the key thread of the evolution of entrepreneurs’ entrepreneurial motivation. (6) Empathy for stakeholders makes entrepreneurs turn into responsible leaders. Responsible leadership is the intermediary/intervention condition in the entrepreneurial process. (7) After becoming a responsible leader, entrepreneurs have taken a series of actions, including implementing CSR behavior to customers, employees, partners, society and other stakeholders. (8) The enterprises in the case have achieved bottleneck crossing and performance improvement. In summary, this paper drew the context relationship between the seven core categories in Table 1 and obtained the entrepreneurial process model of dynamic evolution of entrepreneurial motivation of Chinese entrepreneurs (as shown in Figure 2).

## Model Interpretation

### Entrepreneurial Motivation

#### Egoist Motivation

Egoist motivation refers to a state of motivation whose ultimate goal is to increase its own welfare (Batson, 2008). This study divides egoist motivation into material motivation, personal values and inner satisfaction, risk-taking propensity and achievement motivation:

(1) Material motivation
  Benzing et al. (2009) identified income increase, job security and independence as the main reasons for entrepreneurs to start their own businesses.
  Entrepreneur 3, “The idea of first starting a business was to improve the family’s economic conditions, make the family better, hope to make money, and give the wife and children a better life.”
  Entrepreneur 7, “Because of poverty, when I was in high school, I often used pickles and radishes to fill my stomach. I told my mother: “I must be the master of money, and never be the slave of money.”
(2) Personal values and inner satisfaction
  Some studies suggest that entrepreneurs may be motivated to achieve personal values and intrinsic satisfaction, such as increased job security, a more balanced work-life balance, and increased work autonomy.
  Entrepreneur 11, “Twenty years ago, a woman left her job for medical reasons and returned to her family. But the housewife did not do for long, she and her husband both think that “people are too middle-aged, should not be too idle,” so by chance, her husband “dish” under the low price of three beauty salons on the verge of closure–not to earn back how much money, somehow there is a place, so that she can communicate with her peers.”
(3) Risk-taking propensity
  Other scholars have pointed out that status, reputation and continuous learning are motivations for entrepreneurship. Other studies have looked at the emotion-related motivations, such as passion, happiness, joy, anger and fear, that influence entrepreneurial behavior. However, these motivations are often based on satisfying (or failing to satisfy) ego or risk-taking goals.
  Entrepreneur 8, “Benifits by several years in the industry, he learned everything from the beginning to the end, including manufacturing processes, product inspection, specifications and models, and most importantly, he accumulated a number of industry contacts, which would later lay the foundation for his brand. After full study, I came up with the idea of starting my own business.”
(4) Achievement motivation
  Achievement motivation reflects the need for self-realization based on the intrinsic interest in personal achievement, work itself, responsibility, personal growth and development (Herzberg, 1974).
  Entrepreneur 4: *The core driving force of the first venture is whether it can create greater value.* Belonging to the motivation of achievement in self-interest motivation.

#### Altruistic Motivation

Altruistic motivation refers to a state of motivation whose ultimate goal is to increase the welfare of others (Rim et al., 2016). Altruistic motivation is divided into social responsibility and social mission. Socially responsible entrepreneurs believe that enterprises are not only a tool for them to achieve personal financial goals, but also a tool for them to achieve strong social and environmental values (Choi and Gray, 2008).

In cases,

Entrepreneur 1: *I hope that my experience and knowledge of exercise and weight loss will spread more widely, helping more people to join the army of health and weight loss;*
Entrepreneur 2: *Adhering to the purpose of “introducing high-quality foreign teachers in North America to China, letting every child walk freely on this planet” and “eliminating school districts” to create VIPKid;*
Entrepreneur 3: *Quickly record and share, increase the understanding between people and enhance the unique happiness of each person; help the world to leave as much image as possible, help this era to leave as much memory as possible, and let People seen after a hundred years;*
Entrepreneur 5: *I hope to improve the efficiency of China’s medical system through technology and innovation, so that 1.4 billion people will no longer see a doctor! Make every doctor an expert through AI technology;*
Entrepreneur 10, *Childhood and youth did not have a successful family, which makes entrepreneurs full of yearning for a happy family life, hope to express through the wedding photography industry for a successful marriage and family yearning and desire.*
Entrepreneur 12, *My parents are both village teachers. When I was a child, what I remember vividly is that my father made a hoarse voice when he stood on the platform, and my mother often coughed because of chalk dust and could not sleep at night. Whenever I think of these scenes, My heart is particularly uncomfortable, and I also want to find a solution for them.*

### Bottleneck

Existing studies on the division of enterprise life cycle describe the process of enterprises from small to large, from boom to bust, and rarely discuss the bottleneck. However, the bottleneck period is a critical turning point in the development process of enterprises. Enterprises that break the bottleneck will usher in new growth, while those that are trapped by the bottleneck will decline or even withdraw from the market.

(1) Pain point
  Entrepreneur 10, “An industry is faced with multiple problems: product homogeneity, high customer acquisition cost, low business frequency, low customer loyalty, high turnover rate of technicians, low profit margin and so on.”
(2) Business losses
  Entrepreneur 7, “2012 was the toughest year in corporate history. In the first half of the year, the company’s net loss reached more than 200 million. It was the first time the company had lost money since it was founded, and rising human costs had almost pushed it to the brink of survival.”
(3) Management problems
  Entrepreneur 7, “In terms of management, a series of problems have been exposed, such as stealing business opportunities, forging official seals, signing private contracts and other behaviors harmful to the interests of the company. Some people make false customer confirmation forms for performance.”

### Reasons for Dynamic Evolution of Entrepreneurial Motivation—TP/MTP

Turning point(TP) has been described as a key life event that defines the self-identity of the CSR leader or redefines the self-identity of the CSR entrepreneur. TP narrates a dramatic shift in entrepreneurs’ motivations from self-promotion values to self-transcendence values by redefining their personal values. The personal values of self-transcendence are related to the welfare and interests of others, while the personal values of self-promotion focus on their own interests and domination over others.

The study found that due to the turning point in their life experience, Chinese entrepreneurs changed from self-interested motivation dominated by personal values of self-promotion (focusing on the motivation of wealth and achievement) to altruistic motivation dominated by personal values of self-transcendence (focusing on the welfare and interests of others) (Schwartz, 2010). This study analyzes the role of turning point (TP) in the dynamic evolution of entrepreneurs’ entrepreneurial motivation, and classifies and classifies different forms of TP, which can be divided into four dimensions: life Events in the Present, life events in the past, awareness of unmet social needs and Ideology.

(1) Life events in the present
  “Entrepreneur 1: I have never thought about starting a business. It is entirely because of chance. When a person is thin from 180 to 128 pounds, many people will ask you how thin you are. Otherwise, do you make a small product? If you can help more people, isn’t it a better thing!”;
  “Entrepreneur 4: In 2007, iphone was released. I made a website called apple4.us with a few friends. As the name suggests, it is a group of people who are particularly interested in Apple but have different backgrounds. In a mail group, every day. Some people have raised various questions about Apple, and everyone can only answer some of them; but when you add everyone’s answers together, you will find that this is a new content.”;
  “Entrepreneur 5: “In 2010, Entrepreneur 5’s little nephew got a strange disease. After 10 months, he turned to 7 hospitals and finally went to a large hospital in Shanghai for two synovectomy operations. Finally, under the misunderstanding, Entrepreneur E found the root of the disease: the disease of the child is a complication caused by tuberculosis, and the previous two operations are completely misdiagnosed. At the moment when the nephew came out of the hospital, Entrepreneur E secretly determined: But wherever I can do something for everyone, it is convenient to use their familiar IT technology to make them see a doctor.”
  “Entrepreneur 6: At the end of 2014, Entrepreneur F decided to go on the road again because he took a very smoggy photo on his balcony.”
  “Entrepreneur 10:In the past three years, the number of divorces in China has been on the rise, reaching more than 50% in some big cities. In the wedding photography industry, let Zhou Chen sloppy attitude can see a lot of young people towards marriage, some customers just took photos, haven’t take photos divorced, some customers experienced marriage for many times, and a new set of data show that 28% of customers already pregnant during the filming of a wedding photos–if this part of the family breakdown, so children in single-parent families.”
(2) Life events in the past
  “Entrepreneur 2: When she was 14 years old, she moved from Hebei Province near Beijing to Harbin, the capital of Heilongjiang Province, near the Sino-Russian border in Northeast China. In the new school, she and a math teacher were in their own learning problems. There was a ‘very unpleasant experience’ on her—she said that the conflict prompted her to make up her mind to become an educator and help her children develop a love of learning.”
  “Entrepreneur 3: My grandfather, I have never seen him. He died before I was born. He did not leave photos, and did not leave any diaries or letters. so I have A wish, I hope that everyone can leave more records, which will be seen by future generations. This is a wish of the heart.”
  “Entrepreneur 9 was born in an ordinary small village in Maitreya County, Yunnan. When he was 8 years old, his playmate died of illness due to lack of medicine in the village. The incident haunted Ruan throughout his childhood. At 8 years old, he has not changed the status quo, so that the villagers can have medicine available ambitious, he just blindly hope, if 1 day there is medicine sold in the village, then, his friend may be survived.”
(3) Awareness of unmet social needs
  “Entrepreneur 14: It is estimated that by around 2,050, the number of elderly people in China will peak at 487 million, accounting for 34.9 percent of the total population, the National Office on Aging said. One out of every three people will be elderly! Almost everyone has to face the problem of old age, and every one of us will gradually grow old, old age has become a major social issue.”
(4) Ideology
  “*Entrepreneur 7:* In 2016, I went to Wan Yi Wo in Wang Yangming’s longchang Enlightenment place, where I had my first intimate contact with Mr. Yangming. Then in June, I took 42 senior executives with me to solemnly set our life’s ambition: to be bright.”
  “Entrepreneur 13: To conscience siheyuan teacher gave him a subversive inspiration: technology is important, the heart is fundamental. The one who gets the world is the fundamental avenue of commerce. Modern enterprises cannot underestimate the power of technology, but also must not underestimate the power of human nature. Only technology and products are far from enough, there is no temperature, lack of human care technology, destined to go far!”

### Empathy

There is a lack of research on the division of empathy, and the study of empathy in the field of commercial entrepreneurship is rare. Through the analysis of the founders of the 15 Chinese companies, this research has carried out in-depth research on the empathy of Chinese entrepreneurs. Based on the social network and social cognition theory, this paper divides empathy into colleagues, customers, investors, Four levels of society, the level of entrepreneurship of these four levels is from low to high.

(1) Empathy with customers: *“Entrepreneur 1: Be sure to know where your users are, and then go deep into the real needs of these users, let these users start to like you, willing to feel you. Mainly to practice, Become a user yourself.”;*
(2) Empathy with investors: *“Entrepreneur 1: I usually come up with shortcomings first, such as what kind of people are missing from our team, how much money is lacking, what problems are faced, what are the challenges, etc. Some problems are solved, some have no solutions, and I want to hear their suggestions. Maybe they think that I am a more sincere entrepreneur, and there will be some extra points.”;*
(3) Empathy with the society: “*Entrepreneur 1: KeepKit, hope to go deep into the family, through intelligent hardware, give the family a reason for sports. We will build more homes around the needs of family sports. Sports equipment is passed to every family, allowing each family to experience the charm and happiness of sports.”;*
“Entrepreneur 3: First of all, it is the initial heart, using the market economy method to solve social problems. We believe that recording and sharing is the greatest common denominator of social solutions, and it can make people understand more and make people feel happy. The best solution.”

In the existing research, empathy is mostly applied to social entrepreneurship. This study finds that entrepreneurs of Chinese companies also have empathy. When the interests of colleagues, customers, investors, and society are taken as the responsibility of enterprises, entrepreneurs have relative Level of empathy. As the degree of empathy changes, the trade-off criteria of entrepreneurs making entrepreneurial decisions also change, the degree of empathy is improved, and the interests of others considered by entrepreneurs are more, and more will be obtained. Support, and thus improve the success rate of entrepreneurship.

### CSR Implementation

(1) CSR implementation to employee
  *“Entrepreneur 12:* Establish a happiness school for conscience to spread the family prosperity and career development knowledge to employees and their families, customers, teams and college students.”
(2) CSR implementation to society
  “Entrepreneur 10:The enterprise has released its mission—people’s yearning for a happy marriage is our goal. At the same time announced that the ‘to conscience happy marriage College’ was officially established.”
  *“Entrepreneur 14:* In the next five years, the enterprise will enter 100 cities, build 1,000 nursing homes, enter 10,000 communities, and provide home services to millions of families. Enterprises to achieve a hundred city managers, a thousand deans, ten thousand webmasters, one hundred thousand employees, benefits to ten million families.”
(3) CSR implementation to customer
  “Entrepreneur 7: The company launched the CFO Conscience Institute this year and expects to train tens of thousands of financial and corporate executives by the end of the year.”
  *“Entrepreneur 11:* Although she runs a beauty salon, what she does is not only to beautify and beautify the body, but also to help customers ‘beautiful heart.’ She gradually led more and more women to the construction of their own spiritual quality, the construction of a happy family bright road.”
(4) CSR implementation to supplier
  *“Entrepreneur 12:* Dilute the shares of the chairman and his wife of the group, and establish an online and offline entrepreneurship platform in the oral field for employees, customers and upstream and downstream partners.”

### Entrepreneurial Performance

The entrepreneurial performance of entrepreneurs who experience TP and are driven by altruistic motives is as follows:

Entrepreneur 1: by March 2018, Keep had more than 120 million users, 6.879 billion times of training, 84.1547 million kilometers of running, and more than 45.06 billion kilocaloric calories burned.
Entrepreneur 2: in August 2017, the overall valuation of VIPKid was more than $1 billion, and there were more than 200,000 students and 20,000 teachers on the platform.
Entrepreneur 5: in 2018, the valuation of weimin exceeded $5.5 billion. By June 2017, wechat medical has connected with the information system of more than 2,400 hospitals in 30 provinces in China, and the number of doctors on the platform has exceeded 290,000. In the first half of 2017, the total number of patients served by wechat medical has exceeded 380 million.
Entrepreneur 9: By the end of September 2016, the number of directly operated stores in China has reached 3,877, becoming the enterprise with the largest number of directly operated stores in the domestic pharmaceutical retail industry.

The entrepreneurial performance of entrepreneurs who fail to start a business with egoist motivation and succeed with altruistic motivation is as follows:

Entrepreneur 3: in 2018, kuaishou has become an online community with 100 million daily active users, and the total number of video on kuaishou has exceeded 5 billion;
Entrepreneur 4: by the end of 2017, zhihu had generated 19 million questions, 71 million answers and 250,000 large topics. On average, nearly 30 million active users produce and consume knowledge here every day;
Entrepreneur 6: in 2018, nextev went public in the us stock market, with a market value of $6-8 billion. In December 2017, nextev’s first mass-produced pure electric seven-seat SUVES8 was officially launched and pre-ordered.
Entrepreneur 7: The value of the company has quadrupled in two years.
Entrepreneur 10: Just a year later, not only the staff morale, the performance of its Chongqing headquarters also increased by 23% compared with last year, the turnover rate of marketing staff reduced by half, leaving employees have asked to return to the enterprise.

## Conclusion and Prospects

### Conclusion

First, the entrepreneurial motivation of Chinese enterprises. Previous studies on entrepreneurial motivation of non-social entrepreneurial enterprises mainly focused on individual intrinsic and extrinsic motivation (Deci and Ryan, 1985; Amabile, 1996), intrinsic motivation is mainly achievement motivation, while extrinsic motivation focuses on wealth and status, both of which belong to the category of egoistic motivation. This study found that some entrepreneurs of for-profit entrepreneurial enterprises also have altruistic motives, such as social responsibility and historical mission, and they regard altruistic motives as the primary entrepreneurial motives. This study enriches the dimensions of entrepreneurial motivation of for-profit entrepreneurial enterprises.

Second, the trigger event of the dynamic evolution of entrepreneurial motivation of Chinese entrepreneurs. Study found that the Chinese enterprise entrepreneurs as the triggering event in the life experience, from the emphasis on self-improvement personal values as leading the self-interest motive of motivation and achievement motivation (focused on wealth), into dominated by self beyond the personal values of altruistic motivation (focused on the welfare and interests of the others) (Schwartz, 2010). This study especially analyzes the role of turning point (TP) in the dynamic evolution of entrepreneurial motivation of Chinese entrepreneurs, and classifies different forms of TP into four dimensions: life events in the present, life events in the past, awareness of unmet social needs, Ideology. Although TP has been studied in developmental psychology (for example, Gotlib and Wheaton, 1997; McAdams et al., 2001; Pillemer, 2001) and has conducted limited research in the leadership literature (for example, Bennis and Thomas, 2002; Shamir and Eilam, 2005; Janson, 2008; Ligon et al., 2008; Albert and Vadla, 2009). However, from the perspective of dynamic evolution of entrepreneurial motivation, this study enriched the research field of TP and added new dimensions to TP, found the TP is the evolution of Chinese entrepreneurs of entrepreneurial motivation drivers, TP for entrepreneurs to explain the cause of evolution of entrepreneurial motivation, localization of entrepreneurship in China.

Third, the level of empathy. Based on the social network and social cognition theory, this study divides empathy into four levels: colleague, customer, supplier and society. The degree of empathy of entrepreneurs in these four levels ranges from low to high. In the existing studies, empathy is mostly applied to social entrepreneurship. This study found that entrepreneurs of Chinese enterprises also have empathy, and when they take the interests of colleagues, customers, suppliers and the society as their responsibility, entrepreneurs have a relative level of empathy. With the change of the degree of empathy, the criteria for entrepreneurs to make entrepreneurial decisions also change. With the increase of the degree of empathy, entrepreneurs will take into account the interests of others and get more support when making decisions, so as to improve the entrepreneurial success rate. This study depicts the level of empathy and increases the context of empathy research.

Fourthly, the influence of dynamic evolution of entrepreneurial motivation on the entrepreneurial process of Chinese enterprises. Entrepreneurial motivation of Chinese enterprises entrepreneurs from the initial selfish motives, such as fame motivation, achievement motivation through sublimation TP for altruistic motives in the entrepreneurial process, due to the changes of entrepreneurial motivation, its entrepreneurship and entrepreneurial decision-making also changed, more to consider the welfare of stakeholders, the implementation of CSR behavior, for the welfare of stakeholders, and then get the support of stakeholders, to help enterprises across the bottleneck, performance improvement.

### Theoretical Contribution

This paper adopts grounded theory method to conduct inductive analysis on 15 Chinese entrepreneurs, and constructs an entrepreneurial process model of dynamic evolution of entrepreneurial motivation, which includes egoist motivation, bottleneck, altruistic motivation, TP/MTP, empathy, CSR implementation and entrepreneurial performance. Through the analysis of the internal relationship between these factors, the paper extracts the entrepreneurial process rules of dynamic evolution of Chinese entrepreneurs’ motivation, and reveals the mechanism of dynamic evolution of entrepreneurial motivation. The theoretical contributions of this paper are mainly reflected in the following two aspects: The theoretical contributions of this paper are mainly reflected in the following two aspects:

(1) Enriched and expanded the research results in the field of entrepreneurial motivation of Chinese entrepreneurs. From the perspective of entrepreneurial motivation, this study analyzes the dynamic evolution process of entrepreneurial motivation of Chinese entrepreneurs, extracts the entrepreneurial process rules of dynamic evolution of Chinese entrepreneurs, and provides a new path for enriching and expanding the research on entrepreneurial motivation of Chinese entrepreneurs.
(2) It enriches and complements the study on the triggering events (TP) of the dynamic evolution of entrepreneurial motivation. Using grounded theory, this paper deeply analyzes the reasons for the dynamic evolution of entrepreneurial motivation, and provides empirical evidence for the research on the evolution of localized entrepreneurial motivation in China.

## Limitations and Future Prospects

In terms of research methods, this paper analyzes secondary data. As the research framework is constructed through literature review in advance, although it is convenient to systematically analyze the dynamic evolution process of entrepreneurial motivation, some important information may be omitted. Moreover, the research focuses on the common part of the motivation evolution process between cases, ignores the difference and contrast, and does not put forward the corresponding proposition. In terms of research content, influence factors were not analyzed, and the similarities and differences in the evolutionary process of entrepreneurial motivation between Chinese and foreign entrepreneurs and their reasons were not analyzed in detail. The differences in specific action mechanisms need further discussion. Future research can focus on two aspects:

(1) In view of the depth of the research, detailed research on a certain stage in the dynamic evolution of entrepreneurial motivation, explore its impact on entrepreneurial performance, so as to guide entrepreneurs to improve the level of motivation.
(2) In view of the breadth of the study, compare the dynamic evolution process of entrepreneurial motivation between different regions of China and foreign countries with the active index of entrepreneurial activity in China, find the differences and put forward suggestions and countermeasures from the macro level that are conducive to the evolution of entrepreneurial motivation and the improvement of entrepreneurial performance.

## Data Availability Statement

All datasets generated for this study are included in the article/supplementary material.

## Ethics Statement

An ethics approval was not required as per applicable institutional and national guidelines and regulations. The study is based on a secondary analysis of previously published and publicly available data.

## Author Contributions

YZ was responsible for the manuscript writing. XZ was responsible for the methodology. YQ was responsible for the manuscript revision. All authors listed have made a substantial, direct and intellectual contribution to the work, and approved it for publication.

## Conflict of Interest

The authors declare that the research was conducted in the absence of any commercial or financial relationships that could be construed as a potential conflict of interest.
